# Plasma Macrophage Migration Inhibitory Factor as a Biomarker of Thromboinflammatory Dysregulation in Anti-Neutrophil Cytoplasmic Antibody-Associated Vasculitis

**DOI:** 10.5152/ArchRheumatol.2026.25155

**Published:** 2026-04-03

**Authors:** Tie-Gang Lv, Yuan-Yuan Li, Li-Ping Xu, Jian Hao

**Affiliations:** 1 Division of Radiology, Department of Medicine, Capital Medical University, Beijing Chest Hospital, Beijing, China; 2 Division of Radiology, Department of Medicine, The Affiliated Hospital of Inner Mongolia Medical University, Hohhot, Inner Mongolia Autonomous Region, China; 3 Division of Nephrology, Department of Medicine, The Affiliated Hospital of Inner Mongolia Medical University, Hohhot, Inner Mongolia Autonomous Region, China; 4 Department of Medicine, Inner Mongolia Medical University, Hohhot, Inner Mongolia Autonomous Region, China

**Keywords:** ANCA-associated vasculitis, coagulation, macrophage migration inhibitory factor, MIF, thrombosis

## Abstract

**Background/Aims:**

: Anti-neutrophil cytoplasmic antibody (ANCA)–associated vasculitis (AAV) is an autoimmune disorder characterized by necrotizing inflammation of small vessels. This study investigates the relationship among macrophage migration inhibitory factor (MIF), coagulation parameters, and thrombotic events in AAV.

**Materials and Methods:**

: Plasma and urine samples obtained from 45 AAV patients and 16 healthy controls were analyzed. Then, the MIF levels were quantified via enzyme-linked immunosorbent assay. Afterwards the coagulation markers (prothrombin time (PT), international normalized ratio (INR), activated partial thromboplastin time (APTT), fibrinogen (FIB), fibrin degradation products (FDP), and prothrombin activity (PTA)), renal function (estimated glomerular filtration rate (eGFR)), and disease activity (Birmingham Vasculitis Activity Score (BVAS)) were assessed. Finally, the thrombotic events were radiologically confirmed.

**Results:**

: The plasma MIF levels were significantly elevated in AAV patients when compared to healthy controls (716.35 vs. 293.26 pg/mL, *P *< .05). Beyond demonstrating the associations with disease severity and renal function (which had a positive correlation with BVAS (*r *= 0.391, *P *= .008) and a negative correlation with eGFR (*r *= −0.298, *P *= .047)), MIF further exhibited inverse relationships with high-density lipoprotein cholesterol (*r *= −0.334, *P*=.043). Notably, plasma MIF had significant positive correlations with multiple coagulation parameters, which included PT (*r *= 0.351), INR (*r *= 0.346), APTT (*r *= 0.380), FIB (*r *= 0.374), and FDP (*r *= 0.301) (all, *P *< .05), and a negative correlation with PTA (*r *= −0.346, *P *= .020). Complementing these findings, urinary MIF levels were inversely correlated to thrombin time (*r *= −0.367, *P *= .039), collectively reinforcing the role of MIF in thromboinflammatory dysregulation.

**Conclusion:**

: Although plasma MIF correlates with thromboinflammatory dysregulation, its predictive value for thrombosis warrants validation in larger cohorts.

**Main Points**

- Plasma macrophage migration inhibitory factor (MIF) levels are significantly elevated in patients with active anti-neutrophil cytoplasmic antibody (ANCA)-associated vasculitis (AAV) compared with healthy controls and correlate positively with disease activity assessed by Birmingham Vasculitis Activity Score.
- Higher plasma MIF concentrations are associated with multiple markers of coagulation dysfunction, including prolonged prothrombin time, international normalized ratio and activated partial thromboplastin time, increased fibrinogen and fibrin degradation products, and reduced prothrombin activity.
- Plasma MIF shows inverse correlations with estimated glomerular filtration rate and high-density lipoprotein cholesterol, linking it to both renal impairment and lipid dysregulation in AAV.
- Although plasma MIF strongly reflects thromboinflammatory dysregulation, no significant difference in MIF levels was observed between patients who developed in-hospital thrombotic events and those who did not, likely due to limited statistical power.
- These findings position plasma MIF as a promising biomarker of the inflammation–coagulation axis in ANCA-AAV, warranting validation in larger cohorts for thrombosis prediction and potential therapeutic targeting.

## Introduction

Anti-neutrophil cytoplasmic antibody (ANCA)-associated vasculitis (AAV) comprises granulomatosis with polyangiitis, microscopic polyangiitis, and eosinophilic granulomatosis with polyangiitis systemic autoimmune disorders that target small vessels.^1^^-^^3^ Its defining pathological feature is necrotizing inflammation featuring fibrinoid necrosis in the vessel walls, driving tissue destruction and organ failure. Although these primarily affect the kidneys and lungs, systemic thrombotic complications significantly contribute to morbidity. The prevalence of venous thrombosis in AAV ranges between 5.80% and 30.00%.^4^ Venous thromboembolism (VTE) incidence is markedly elevated during active disease, with increased relative risk *vs.* remission or healthy controls.^5^ This thrombophilic phenotype underscores the necessity for the mechanistic exploration of coagulation dysregulation in AAV pathophysiology.

Macrophage migration inhibitory factor (MIF) exhibits significantly elevated plasma concentrations in patients with AAV when compared to those in remission or healthy controls, underscoring its contribution to disease pathogenesis. Mechanistically, MIF primes neutrophils by promoting ANCA antigen translocation to the cell membrane, thereby enhancing its susceptibility to ANCA-mediated activation. This process triggers respiratory bursts and degranulation, amplifying vascular inflammation.^6^ Beyond its role in leukocyte recruitment, MIF serves as a critical nexus between inflammation and coagulation pathways. It mediates inflammatory cell infiltration and accelerates plaque progression within the vascular wall.^7^^,^^8^ Notably, MIF interacts with CXCR7 to modulate platelet survival and thrombotic potential, both in vitro and in vivo, suggesting its regulatory role in thrombosis and inflammation.^9^ Unlike other platelet-derived chemokines, MIF exhibits delayed secretion kinetics and unique autocrine/paracrine signaling properties. Importantly, MIF acts as a major platelet-derived chemotactic factor with clot-modulating effects, implicating its relevance in inflammatory diseases such as atherosclerosis.^10^

Although MIF has been well-established in AAV pathogenesis, particularly through mediating ANCA-induced neutrophil activation, degranulation and reactive oxygen species production are the well-established drivers of vascular inflammation in AAV.^11^^,^^12^ However, its specific contributions to AAV-associated hypercoagulability remain poorly understood. Clinical observations have revealed that AAV patients maintain markedly elevated thrombotic risk, not only during active disease, but also in remission, as evidenced by persistent coagulation abnormalities.^13^

Although the highest incidence of VTE occurs during active disease and early disease course, emerging data have demonstrated increased thrombogenic potential in peripheral blood samples, even from patients in clinical remission.^14^ This growing body of evidence supports the existence of a sustained hypercoagulable state in AAV, highlighting the urgent need for studies that evaluate tailored anticoagulation strategies to mitigate thromboembolic complications in this population.

Thus, the investigators hypothesized that plasma MIF levels are elevated in active AAV and correlate with both disease activity and coagulation dysfunction, thereby serving as a biomarker of thromboinflammatory dysregulation.

## Materials and Methods

### Study Population

The present prospective study enrolled 45 treatment-naïve AAV patients (18 male and 27 female patients; at enrollment, no anticoagulants (including heparin) were administered prior to or during sample collection), who were hospitalized at the Department of Nephrology, The Affiliated Hospital of Inner Mongolia Medical University, between November 2021 and December 2024. The cohort comprised 43 myeloperoxidase (MPO)-ANCA-positive and 2 proteinase 3 (PR3)-ANCA-positive cases, and all cases met the 2012 Chapel Hill Consensus Conference diagnostic criteria.^15^ Exclusion criteria: (1) active severe infections (particularly sepsis or urinary tract infections); (2) other glomerulonephritis types (e.g. lupus nephritis); (3) comorbid autoimmune diseases (e.g. rheumatoid arthritis); (4) present therapeutic anticoagulation. A total of 16 age- and gender-matched healthy volunteers served as the healthy controls. The present study protocol was approved by the Ethics Committee of the Affiliated Hospital of Inner Mongolia Medical University l (Approval no: YKD202001155, Approval date: Apr 07, 2020), and written informed consent was obtained from all participants.

### Standardized Biospecimen Collection

Fasting venous blood (5 mL) was collected between 6 and 8 am within 24 hours of admission, along with 10 mL of midstream clean-catch morning urine. All samples were processed within 30 minutes at room temperature under standardized conditions: blood samples were collected into sodium citrate anticoagulant tubes and centrifuged at 3000 × g for 20 minutes at 20-24°C without brake to obtain platelet-poor plasma (platelet count <10 × 10^9^/L), while urine samples were immediately aliquoted into sterile Eppendorf tubes. Then, the processed aliquots were stored at −20°C with a strict limitation to ≤2 freeze-thaw cycles in order to preserve the sample integrity. Key biomarkers, including MIF (Cat# bsk11047; Bioss, Beijing, China) and coagulation parameters (prothrombin time (PT), activated partial thromboplastin time (APTT), fibrinogen (FIB), and fibrin degradation products (FDP)), were analyzed in batches within 3 months of collection in order to ensure analytical consistency. Absorbance (450 nm) was measured using the SpectraMax i3x microplate reader (Molecular Devices). The intra- and inter-assay coefficients of variation were <8% and <12%, respectively. All values fell within the standard curve range (8.23-6000.00 pg/mL), and the results were reported in pg/mL.

### Baseline Clinical Characteristics Data

The comprehensive baseline clinical characteristics were systematically collected from medical records, which included the following: demographic information (age and gender), detailed medical history, admission physical examination findings, and present treatment regimens.

### Laboratory and Imaging Data

The comprehensive laboratory test included the following: complete blood count (hemoglobin (HGB), white blood cell count (WBC), and platelet count (PLT)), coagulation profile (PT, international normalized ratio (INR), APTT, thrombin time (TT), FIB, FDP, and D-dimer), inflammatory markers (erythrocyte sedimentation rate (ESR), C-reactive protein (CRP), and complement C3/C4), liver function tests (alanine aminotransferase, aspartate aminotransferase, and albumin (ALB)), metabolic panel (total cholesterol (TCH), high-density lipoprotein cholesterol (HDL-C), low-density lipoprotein cholesterol, triglycerides, and glucose), and renal function parameters (serum creatinine (SCr)). The coagulation tests were performed on citrate-anticoagulated plasma, and TT was measured using standard clinical assays without exogenous heparin interference. D-dimer was measured using the latex-enhanced immunoturbidimetric assay (Sekisui Diagnostics, Burlington, MA, USA). The results are reported in μg/mL FEU (FIB equivalent units), with the laboratory reference range of 0-0.55 μg/mL FEU. All patient values were above the upper limit of normal (median: 3.42 μg/mL FEU, interquartile range (IQR): 1.68-6.89 μg/mL FEU). The estimated glomerular filtration rate (eGFR) was calculated using the Modification of Diet in Renal Disease formula: 186 × (SCr in mg/dL) × age (years) × 0.742 (if female).

The imaging studies included vascular ultrasound (lower extremity, carotid, intracranial, and renal vessels), angiography (coronary and cerebral), magnetic resonance angiography, and other relevant modalities. All laboratory tests and imaging studies were performed and interpreted by the certified clinical laboratory and radiology department of the hospital of the investigators, respectively.

### Statistical Analysis

All statistical analyses were performed using SPSS version 27.0 (IBM SPSS Corp.; Armonk, NY, USA). Normally distributed continuous variables are expressed as mean ± standard deviation and compared by independent samples *t*-test. Non-normally distributed continuous variables are presented as median (IQR) and analyzed by Mann-Whitney *U*-test. Categorical data are expressed as percentage and compared by chi-square test or Fisher’s exact test, as appropriate. The correlation analysis was conducted using Pearson’s test for normally distributed variables. The correlation coefficients were reported with 95% CI estimated via 1000 bootstrap resamples. The sample size (n) was specified for each analysis. The correlation analysis between plasma MIF and multiple coagulation parameters was considered exploratory. Thus, no correction for multiple comparisons (e.g., false discovery rate) was applied, and the *P*-values were reported unadjusted. A 2-tailed *P*-value of <.05 was considered statistically significant.

## Results

### Clinical Characteristics of Anti-Neutrophil Cytoplasmic Antibody–Associated Vasculitis Patients

The present study included 45 AAV patients with a median age of 68.00 years (IQR: 60.00-73.50). Among these patients, 18 were male (40%) and 27 were female (60%). Furthermore, the plasma MIF levels, which were measured by enzyme-linked immunosorbent assay (ELISA), were significantly elevated (median: 716.35 pg/mL, IQR: 446.73-1415.99). Moreover, among these patients, 13 (28.9%) presented with anuria (indicating end-stage renal disease (ESRD)), and the measured urinary MIF level was 148.78 pg/mL (IQR: 60.12-398.22) by ELISA.

The comparative analysis revealed significant differences between AAV patients and healthy controls, in both plasma and urine parameters. For the plasma biomarkers, AAV patients had significantly higher levels of WBC, PT, INR, FIB, SCr, and TCH, and significantly lower HGB, prothrombin activity (PTA), and ALB, when compared to healthy controls (all, *P *<. 05). Similar patterns were observed in the urinary analysis of AAV patients with available urine data. Notably, the 2 groups had no significant differences in age or gender distribution (*P *> .05). The complete baseline clinical and laboratory characteristics are presented in Table 1.

### Elevated Plasma Migration Inhibitory Factor Is Associated to Disease Activity

**Figure 1.**
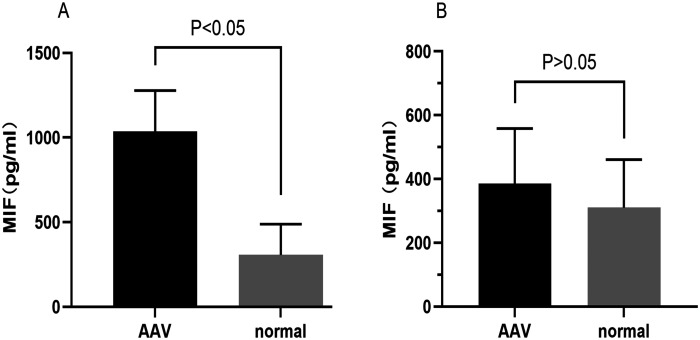
Comparative analysis of plasma (panel A) and urinary (panel B) macrophage migration inhibitory factor (MIF) levels in anti-neutrophil cytoplasmic antibody-associated vasculitis (AAV) patients vs. healthy controls.

**Figure 2.**
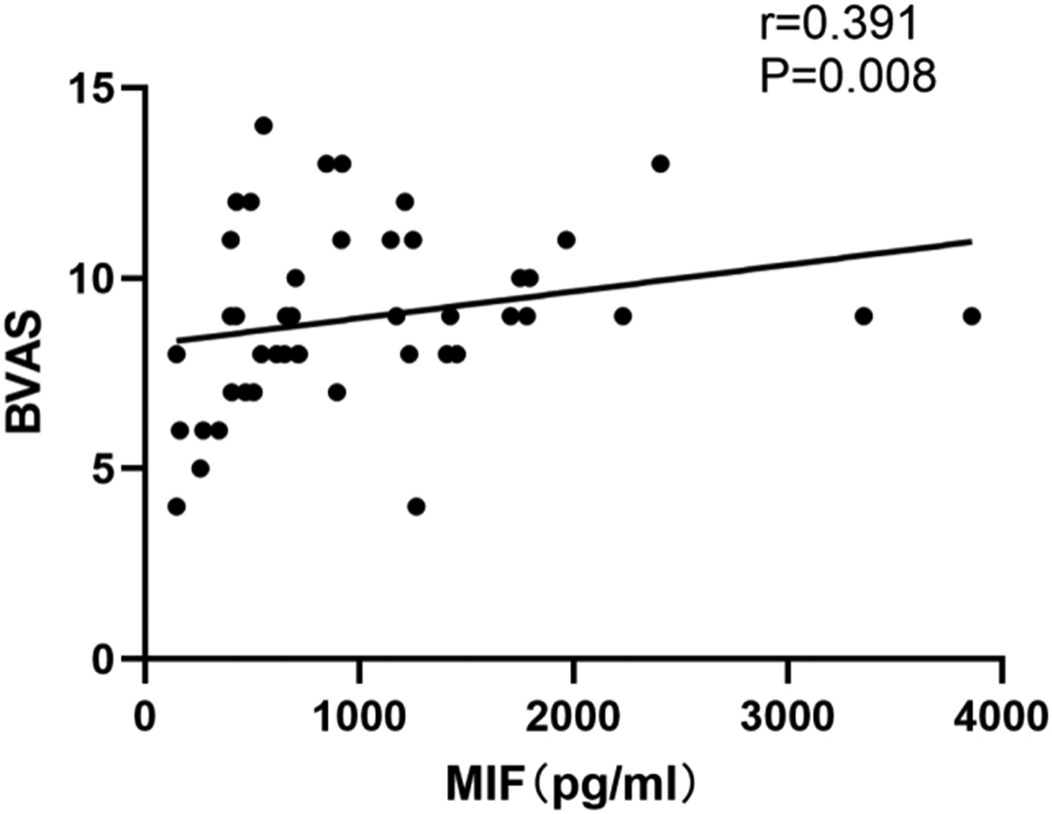
The plasma macrophage migration inhibitory factor (MIF) levels were significantly positively correlated to the Birmingham Vasculitis Activity Score (BVAS) in anti-neutrophil cytoplasmic antibody-associated vasculitis patients.

**Figure 3.**
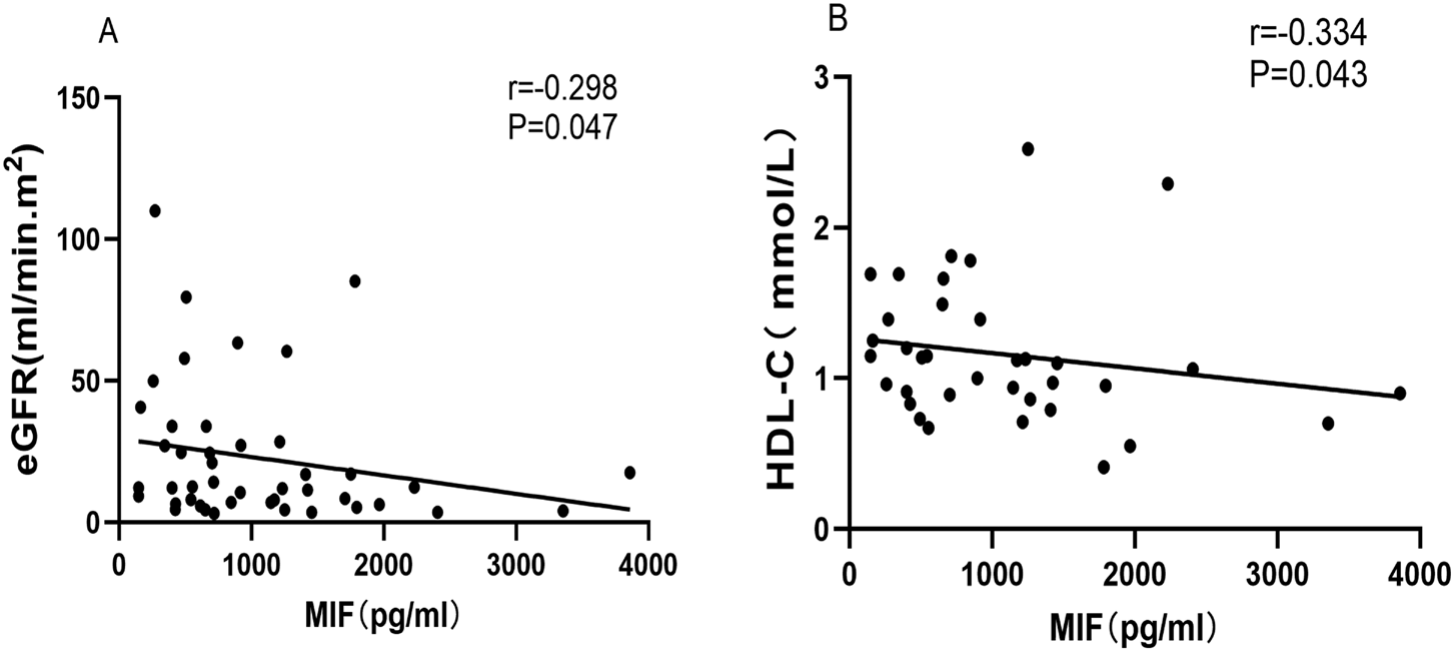
Plasma macrophage migration inhibitory factor (MIF) levels were significantly inversely correlated to the estimated glomerular filtration rate (eGFR, panel A) and high-density lipoprotein cholesterol (HDL-C, panel B) in anti-neutrophil cytoplasmic antibody-associated vasculitis patients.

The plasma MIF levels exhibited a significant 2.4-fold elevation in AAV patients vs. healthy controls (*P *< .05, Figure 1A), while the urinary MIF concentrations remained comparable between groups (Figure 1B). The correlation analysis revealed that the plasma MIF levels were positively associated with disease activity (Birmingham Vasculitis Activity Score (BVAS): *r = *0.391, *P = *.008; Figure 2), but inversely correlated with renal function (eGFR: *r *= −0.298, *P *= .047; Figure 3A) and HDL-C levels (*r = −*0.334, *P *= .043; Figure 3B). No significant correlation was observed between the D-dimer and MIF concentrations in either the plasma or urine obtained from AAV patients: plasma MIF vs. D-dimer (*r *= 0.262, *P *= .080); urinary MIF vs. D-dimer (*r *= 0.043, *P *= .814).

### Plasma Migration Inhibitory Factor Is Correlated to Coagulopathy Markers

**Figure 4.**
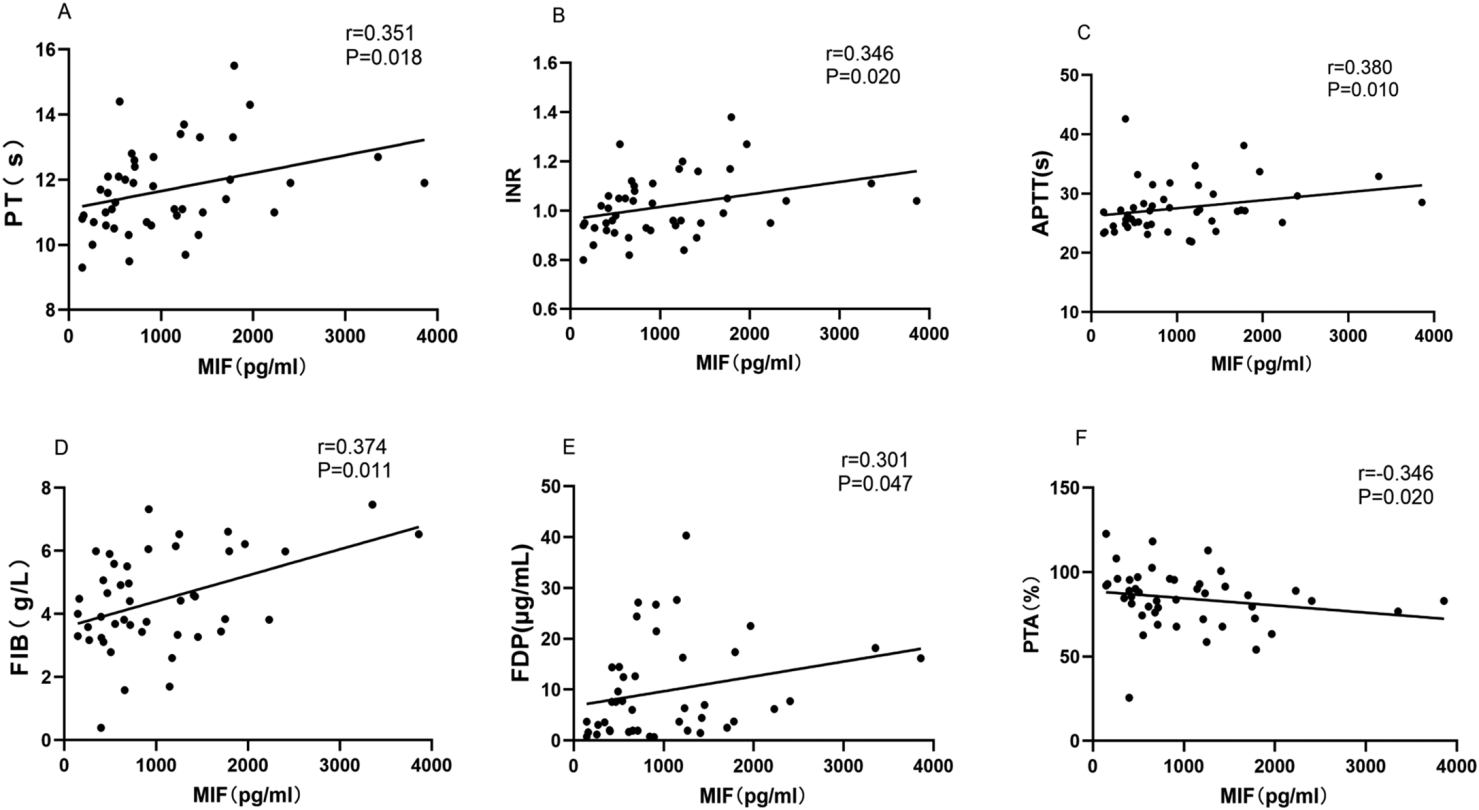
Association analysis between the plasma macrophage migration inhibitory factor (MIF) levels and coagulation parameters in anti-neutrophil cytoplasmic antibody-associated vasculitis patients: prothrombin time (PT, panel A), international normalized ratio (INR, panel B), activated partial thromboplastin time (APTT, panel C), fibrinogen (panel D), fibrin degradation products (FDP, panel E), and prothrombin activity (PTA, panel F).

In AAV patients, the plasma MIF levels had statistically significant positive correlations with prolonged PT (*r *= 0.351, *P *= .018; Figure 4A), elevated INR (*r *= 0.346, *P *= .020; Figure 4B), extended APTT (*r *= 0.380, *P *= .010; Figure 4C), increased FIB (*r *= 0.374, *P *= .011; Figure 4D), and elevated FDP (*r *= 0.301, *P *= .047; Figure 4E). Conversely, an inverse correlation was observed between the MIF concentrations and PTA (*r *= −0.346, *P *= .020; Figure 4F).

### The Lack of Significant Association Between Plasma/Urinary Migration Inhibitory Factor Levels and In-hospital Thrombotic Events

The correlation analysis of urinary MIF levels and clinical parameters in AAV patients revealed no significant associations with general clinical markers. As detailed in Table 2, the assessment of coagulation parameters revealed a statistically significant inverse correlation between urinary MIF and TT (*r *= −0.367, *P *= .039).

The observed correlations between plasma MIF and coagulation parameters prompted the analysis of thrombotic events. Among the 45 AAV patients, 7 (15.6%) patients developed thrombotic complications during hospitalization: 6 patients had deep vein thrombosis (DVT) and 1 patient had cerebral infarction. In order to evaluate the potential associations between MIF levels and thrombotic risk, the patients were stratified into 2 groups: thrombotic event-positive (n = 7) and thrombotic event-negative (n = 38). The plasma MIF concentration was 700.82 (504.52-1212.62) pg/mL in the thrombotic group vs. 780.49 (424.33-1431.80) pg/mL in the non-thrombotic group (U = 132.00, *P* = .975). Similarly, the urinary MIF level was 329.59 (206.16-439.06) pg/mL vs. 88.15 (49.94-367.60) pg/mL (U = 42.00, *P* = .082). There were no statistically significant differences observed in either of the comparisons.

## Discussion

The present study revealed moderate correlations between plasma MIF levels and coagulation dysfunction in AAV, including PT/APTT, hyperfibrinogenemia, and elevated FDP. These findings establish plasma MIF as a biomarker of thromboinflammatory dysregulation in AAV, concurrently reflecting inflammatory activity and coagulation dysfunction. Although plasma MIF exhibited its potential predictive value for thrombotic risk through its association with coagulation parameters, this requires validation in larger cohorts, given the underpowered thrombotic subgroup analysis. Targeting the MIF-thromboinflammatory axis represents a rational therapeutic strategy to mitigate coagulation complications in AAV.

Anti-neutrophil cytoplasmic antibody–associated vasculitis predominantly affects middle-aged and elderly populations, with heterogeneous clinical manifestations frequently involving renal and pulmonary systems that may progress to ESRD.^16^^,^^17^ Substantial evidence has indicated significantly elevated thrombotic risk in AAV patients, including DVT, pulmonary embolism, myocardial infarction, and cerebral infarction secondary to cerebral artery thrombosis.^18^^,^^19^ Emerging clinical and experimental data have implicated MIF as a pivotal regulator of coagulation pathways in AAV pathogenesis.^20^ Therefore, the investigators hypothesized that MIF potentiates thrombosis risk in AAV through the synergistic promotion of procoagulant activity and inflammatory responses.

Accumulating evidence has confirmed that MIF is highly expressed by intrinsic renal cells, infiltrating macrophages, and T lymphocytes, with elevated levels implicated in the pathogenesis of immune-mediated nephropathies, acute kidney injury, and other renal disorders.^21^^,^^22^ Notably, plasma MIF levels in active AAV significantly exceed those in healthy controls and remission-phase AAV patients,^12^ correlating to disease activity.^23^ This is a finding corroborated by the present study.

Although the urinary MIF levels in the present AAV cohort numerically increased when compared to healthy controls, this difference lacked statistical significance. This contrasts with reports that revealed elevated urinary MIF in proliferative glomerulonephritis (including crescentic forms) vs. non-proliferative disease and healthy subjects, in which the levels correlated to renal injury severity.^24^ This negative finding may reflect the limited statistical power: the post-hoc analysis indicated only 32% power to detect a 30% MIF elevation (*α* = 0.05). Future multi-center studies with larger cohorts are warranted to validate the predictive utility of MIF for thrombosis. Importantly, the present data established MIF as a biomarker of thromboinflammatory dysregulation, rather than confirming a causal role.

**Supplementary Figure 1.**
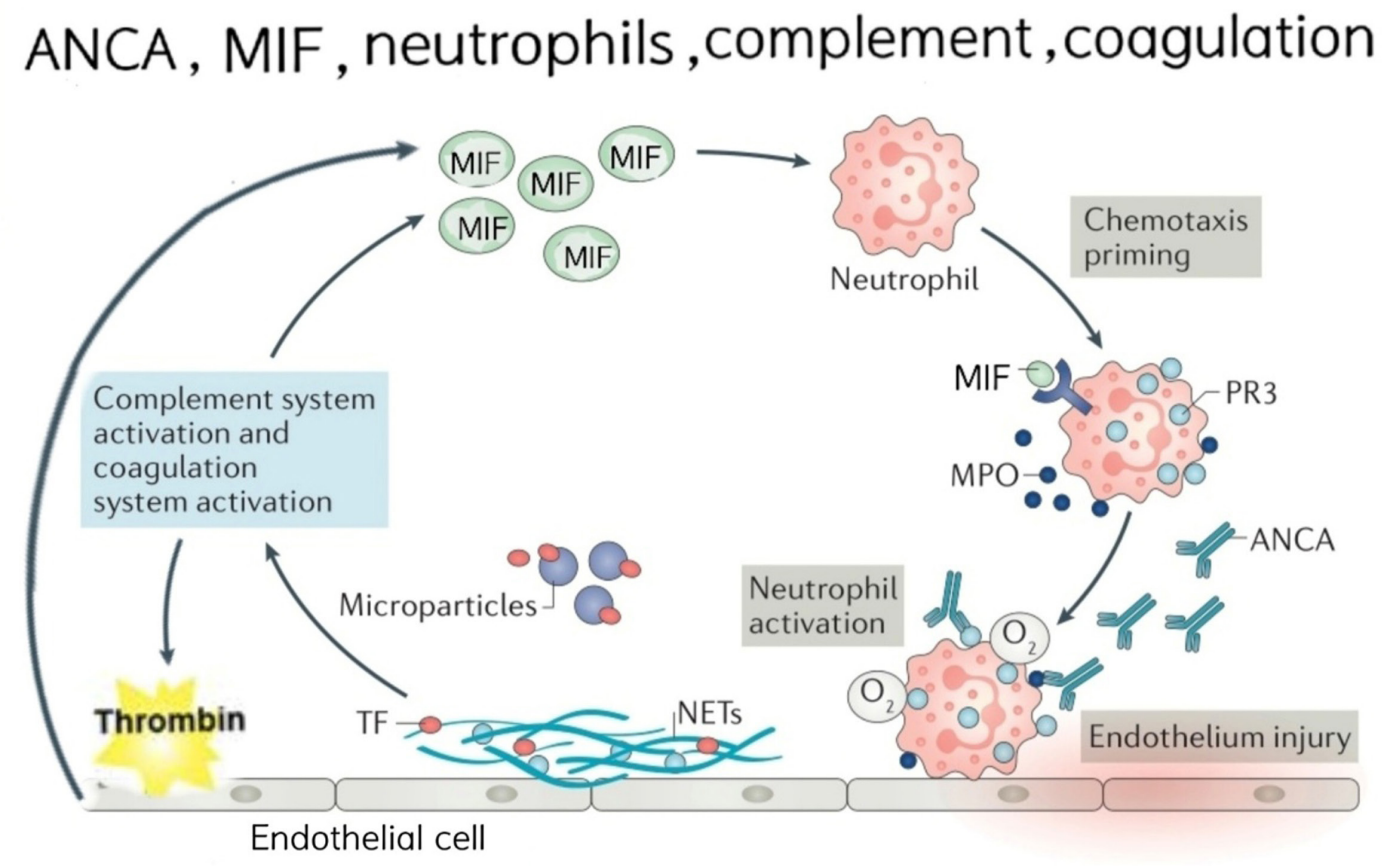
Schematic representation of the proposed pathogenic amplification loop linking migration inhibitory factor (MIF), neutrophil activation, anti-neutrophil cytoplasmic antibody (ANCA), NETosis, tissue factor (TF) release, and coagulation in ANCA-associated vasculitis (AAV). Elevated circulating or tissue MIF binds to CXCR4/CD74 on resting neutrophils, and primes these. Upon subsequent encounter with ANCA autoantibodies, primed neutrophils undergo full activation via Fcγ receptors and PR3/MPO engagement. Activated neutrophils release TF-bearing microparticles, and form neutrophil extracellular traps (NETs) decorated with TF. These procoagulant structures trigger extrinsic coagulation pathway activation and thrombin generation. In turn, thrombin further upregulates the endothelial MIF release, and activates platelets, which serve as both an important source of circulating MIF and a target of MIF-mediated activation. The resulting complement activation, endothelial injury, and microthrombosis amplify the inflammatory-thrombotic cycle characteristic of active AAV. The model integrates clinical MIF-coagulation correlations and experimental evidence, showing the ANCA-induced TF and NET release.

Migration inhibitory factor propagates a pathogenic thromboinflammatory cycle in AAV by simultaneously driving neutrophil activation via enhanced ANCA antigen translocation and coagulation initiation,^12^ thereby amplifying the ANCA-mediated activation and release of tissue factor-bearing microparticles and neutrophil extracellular traps.^25^ These procoagulant effectors initiate the extrinsic coagulation cascade, driving thrombin generation and fibrin formation, and mechanistically accounting for the observed positive correlations between MIF and PT, INR, APTT, FIB, and FDP. In turn, thrombin upregulates endothelial MIF via PAR-1 signaling, while activated platelets both release the stored MIF and respond to it through CXCR7, prolonging the survival and thrombotic potential.^26^^-^^28^ This bidirectional amplification sustains the self-perpetuating MIF-thromboinflammatory cycle that underlies persistent hypercoagulability in AAV (Supplementary Figure 1).

The present data supports the involvement of this mechanism, demonstrating a significant correlation between plasma MIF and FIB alongside dysregulated coagulation parameters (e.g. prolonged PT/APTT and elevated FDP). These alterations collectively reflect the sustained thromboinflammatory activity in AAV. For future clinical translation, plasma MIF monitoring (particularly when combined with D-dimer) may enhance thrombotic risk stratification in high-risk AAV subgroups (e.g. PR3-ANCA+, hypoalbuminemia, or eGFR <30 mL/min), pending its validation in larger cohorts with adequate statistical power for thrombosis prediction.

Collectively, these data positions plasma MIF not only as a marker of AAV activity but also as a central node in the inflammation-lipid-coagulation triad that drives both renal and cardiovascular morbidity. Future studies should incorporate serial MIF and lipid profiling during remission induction and determine whether the MIF blockade (e.g. via small-molecule inhibitors or anti-MIF antibodies) can restore HDL function and reduce thrombotic risk in AAV. It is noteworthy that none of the patients in the present treatment-naïve cohort received statin therapy, precluding the confounding by lipid-lowering agents. Thus, the significant inverse correlation between plasma MIF and HDL-C reflects a biologically plausible, statin-independent association.

Although VTE is a well-documented complication in AAV, its pathogenesis remains incompletely characterized, with identified risk factors, including PR3-ANCA positivity, hypoalbuminemia, renal impairment, and advanced age.^29^ Although the present analysis of the 45 AAV patients revealed no significant difference in MIF levels between the thrombotic and non-thrombotic subgroups, which was potentially due to the limited event incidence during hospitalization, and the dynamic MIF fluctuations uncaptured by single-point admission sampling, patients who developed thrombosis had significantly elevated WBC, PTA, D-dimer, and FDP, indicating baseline prothrombotic and proinflammatory states. This aligns with the evidence of persistent coagulation abnormalities (e.g., elevated D-dimer in 40% of remission patients), reflecting the disease-phase-dependent hemostatic dysregulation primarily driven by the intrinsic coagulation pathway.^30^ Although the present study established significant correlations between MIF and multiple coagulation parameters, the complex bidirectional interplay within the MIF-thromboinflammatory axis warrants further mechanistic investigation.

The present study revealed the significant inverse correlation between plasma MIF levels and eGFR, indicating that MIF elevation can reflect renal impairment in AAV, although earlier chronic kidney disease studies have attributed elevated MIF to oxidative stress/endothelial activation, rather than renal function.^31^ Furthermore, recent evidence supports the present findings, in which the low molecular weight (12.50 kDa) of MIF facilitates glomerular filtration, suggesting that the correlation stems from the combined effects of impaired renal clearance and disease-driven overproduction.^32^ The potential of plasma MIF as a biomarker for eGFR decline and treatment response warrants longitudinal validation. Urinary MIF had limited coagulation parameter associations (merely a positive correlation with TT), which was likely confounded by renal excretion dynamics.

The present study identified a significant inverse correlation between plasma MIF and HDL-C levels in AAV. Beyond its cholesterol reverse transport function, high-density lipoprotein (HDL) exerts anti-inflammatory, antioxidant, and endothelial-protective effects.^33^^,^^34^ Although HDL-C concentration does not fully reflect HDL functionality, its clinical measurement remains inversely associated with atherosclerotic cardiovascular risk. These present findings align with established MIF-cardiovascular event associations,^35^ suggesting that elevated MIF may portend increased thrombotic cardiovascular risk in AAV, including myocardial infarction.^36^^,^^37^ Notably, dyslipidemia in vasculitis correlates with endothelial damage, in which hypertriglyceridemia predicts ESRD progression in MPA via pro-fibrotic/inflammatory signaling and complement/coagulation cascade activation.^38^

The study limitations include the following: (1) the present study had a single-center design with limited sample size; (2) there was a lack of serial MIF measurements during thrombotic events, given the rapid secretion of MIF during neutrophil activation; (3) undetected coagulation factors restricted mechanistic exploration; (4) the reported correlations between plasma MIF and coagulation parameters were unadjusted and may have been influenced by confounding factors, such as renal impairment (which affects MIF clearance) and systemic inflammation (reflected by BVAS, WBC, and CRP), necessitating multivariable adjustment in future studies. Despite the null correlations with ESR/CRP or complement, the inflammation-endothelium-coagulation-complement nexus remains crucial. The longitudinal tracking revealed 6 thrombosis cases within 2 years, underscoring the imperative for future mechanistic and preventative studies.

It is noteworthy that the present cohort was heavily skewed towards MPO-ANCA positivity (43/45, 96%), reflecting a predominantly renal-involved, MPO-predominant AAV population. This serotype distribution limits the generalizability to PR3-ANCA-positive disease, which is independently associated with heightened VTE risk.^39^ The MIF-thromboinflammatory associations observed in the present study may thereby be most relevant to MPO-driven pathophysiology, and the relationship between MIF and thrombosis can differ by ANCA serotype. Future studies should be adequately powered to stratify analyses by MPO- vs. PR3-ANCA status, in order to clarify the serotype-specific mechanisms and biomarker utility.

Plasma MIF serves as a biomarker that reflects disease activity in AAV. Beyond its inverse correlations with renal function and HDL-C levels, MIF has significant associations with coagulation dysfunction. These findings collectively establish MIF as an indicator of thromboinflammatory dysregulation in AAV, suggesting a potential role in its pathogenesis that warrants mechanistic investigation.

## Figures and Tables

**Table 1.** Baseline Characteristics of AAV Patients vs. Healthy Controls

	**AAV (** **n ** **= 45)**	**Healthy Controls (** **n ** **= 16)**	*t*/*x^2^*/*z*	*P*
Gender (male/female)	18/27	8/8	0.483	.487
Age	68.00 (60.00, 73.50)	64.50 (59.00, 68.00)	−1.790	.074
BVAS	8.98 ± 2.34	0 (0, 0)	−5.987	<.001
HGB (g/L)	99.38 ± 24.36	152.81 ± 13.89	−8.281	<.001
WBC (10^9^/L)	8.09 (5.71, 11.20)	5.92 (5.08, 6.87)	−2.836	.005
PLT (10^9^/L)	218.36 ± 108.07	226.63 ± 56.35	−0.386	.701
PT (s)	11.60 (10.75, 12.65)	10.85 (10.53, 11.00)	−2.511	.012
INR	1.01 (0.94, 1.11)	0.95 (0.91, 0.95)	−2.424	.015
PTA (%)	84.34 ± 17.21	93.37 ± 5.72	−3.075	.003
APTT (s)	27.00 (24.70, 29.30)	27.25 (25.98, 27.98)	−0.336	.737
FIB (g/L)	4.43 ± 1.55	2.72 ± 0.55	6.366	<.001
TT (s)	16.90 (16.30, 17.90)	16.95 (16.35, 17.45)	−0.246	.806
SCr (μmol/L)	389.00 (185.00, 631.00)	54.50 (48.50, 71.00)	−5.673	<.001
ALB (g/L)	32.13 ± 7.11	44.64 ± 3.49	−9.055	<.001
ALT (U/L)	14.20 (9.48, 28.00)	22.50 (16.53, 28.33)	−1.730	.084
AST (U/L)	17.40 (13.00, 22.35)	16.40 (14.60, 19.63)	−0.025	.980

AAV, anti-neutrophil cytoplasmic antibody-associated vasculitis; ALB, albumin; ALT, alanine aminotransferase; APTT, activated partial thromboplastin time; AST, aspartate aminotransferase; BVAS, the Birmingham Vasculitis Activity Score; FIB, fibrinogen; HGB, hemoglobin; INR, international normalized ratio; PLT, platelet count; PT, prothrombin time; PTA, prothrombin activity; SCr, serum creatinine; TT, thrombin time; WBC, white blood cell count.

**Table 2.** Correlation Analysis Between Urinary MIF Levels and Coagulation Parameters in AAV Patients

	*r*	*P*
TT (s)	−0.367	.039
PLT (10^9^/L)	−0.041	.826
PT (s)	0.063	.732
APTT (s)	−0.091	.620
FIB (g/L)	0.089	.628
D-D (μg/mL)	0.043	.814
FDP (μg/mL)	0.079	.673

AAV, anti-neutrophil cytoplasmic antibody-associated vasculitis; APTT, activated partial thromboplastin time; D-D, D-dimer; FDP, fibrin degradation products; FIB, fibrinogen; MIF, migration inhibitory factor; PLT, platelet count; PT, prothrombin time; TT, thrombin time.

## Data Availability

The data that support the findings of this study are available on request from the corresponding author.

## References

[1] Trivioli G Casal Moura M Kronbichler A Advances in the treatment of ANCA-associated vasculitis. Nat Rev Rheumatol. 2025;21(7):396 413. (doi: 10.1038/s41584-025-01266-1) 40473820

[2] Jayne D. Emerging targeted therapies in ANCA-associated vasculitis. Rheumatol (Oxf Engl). 2025;64(suppl 1):i15 i18. (doi: 10.1093/rheumatology/keae663) 40071424

[3] Chen SF Li ZY Zhao MH Anti-neutrophil cytoplasmic antibody-associated vasculitis in china: epidemiology, management, prognosis, and outlook. Kidney Dis (Basel). 2024;10(5):407 420. (doi: 10.1159/000540514) 39430288 PMC11488837

[4] Emmi G Silvestri E Squatrito D Thrombosis in vasculitis: from pathogenesis to treatment. Thromb J. 2015;13(1):15. (doi: 10.1186/s12959-015-0047-z) PMC4399148 25883536

[5] Zhu K Lv F Hou X Thrombosis in vasculitis: an updated review of etiology, pathophysiology, and treatment. Heliyon. 2024;10(12):e30615. (doi: 10.1016/j.heliyon.2024.e30615) PMC11225688 38975109

[6] Hao J Lv T-G Wang C Macrophage migration inhibitory factor contributes to anti-neutrophil cytoplasmic antibody-induced neutrophils activation. Hum Immunol. 2016;77(12):1209 1214. (doi: 10.1016/j.humimm.2016.08.006) 27544048

[7] Grieb G Merk M Bernhagen J Macrophage migration inhibitory factor (MIF): a promising biomarker. Drug News Perspect. 2010;23(4):257 264. (doi: 10.1358/dnp.2010.23.4.1453629) 20520854 PMC3131110

[8] Wen Y Cai W Yang J Targeting macrophage migration inhibitory factor in acute pancreatitis and pancreatic cancer. Front Pharmacol. 2021;12(1):638950. (doi: 10.3389/fphar.2021.638950) PMC7992011 33776775

[9] Chatterjee M Borst O Walker B Macrophage migration inhibitory factor limits activation-induced apoptosis of platelets via CXCR7-dependent Akt signaling. Circ Res. 2014;115(11):939 949. (doi: 10.1161/CIRCRESAHA.115.305171) 25266363

[10] Wirtz TH Tillmann S Strüßmann T Platelet-derived MIF: a novel platelet chemokine with distinct recruitment properties. Atherosclerosis. 2015;239(1):1 10. (doi: 10.1016/j.atherosclerosis.2014.12.039) 25561410

[11] Wendt M Börjesson O Avik A Macrophage migration inhibitory factor (MIF) and thyroid hormone alterations in antineutrophil cytoplasmic antibody (ANCA)-associated vasculitis (AAV). Mol Med. 2013;19(1):109 114. (doi: 10.2119/molmed.2012.00352) 23552723 PMC3667215

[12] Becker H Maaser C Mickholz E Relationship between serum levels of macrophage migration inhibitory factor and the activity of antineutrophil cytoplasmic antibody-associated vasculitides. Clin Rheumatol. 2006;25(3):368 372. (doi: 10.1007/s10067-005-0045-9) 16391884

[13] Busch MH Ysermans R Aendekerk JP The intrinsic coagulation pathway plays a dominant role in driving hypercoagulability in ANCA-associated vasculitis. Blood Adv. 2024;8(5):1295 1304. (doi: 10.1182/bloodadvances.2023011937) 38175623 PMC10918483

[14] Stacey HL Francis L Smith RM Practical management of ANCA-associated vasculitis: A clinician’s perspective. Glomerular Dis. 2025;5(1):84 102. (doi: 10.1159/000543159) 39991192 PMC11845170

[15] Jennette JC Falk RJ Bacon PA 2012 revised International Chapel Hill Consensus Conference Nomenclature of Vasculitides. Arthritis Rheum. 2013;65(1):1 11. (doi: 10.1002/art.37715) 23045170

[16] Floege J Jayne DRW Sanders JF Executive summary of the KDIGO 2024 Clinical Practice Guideline for the Management of ANCA-Associated Vasculitis. Kidney Int. 2024;105(3):447 449. (doi: 10.1016/j.kint.2023.10.009) 38388147

[17] Merkel PA George MD Yue H Safety of avacopan for the treatment of antineutrophil cytoplasmic antibody-associated vasculitis: combined data from three clinical trials. ACR Open Rheumatol. 2025;7(4):e70001. (doi: 10.1002/acr2.70001) PMC11973930 40192160

[18] Jin Y Wang F Tang J Low platelet count at diagnosis of anti-neutrophil cytoplasmic antibody-associated vasculitis is correlated with the severity of disease and renal prognosis. Clin Exp Med. 2024;24(1):70. (doi: 10.1007/s10238-024-01333-z) PMC10997538 38578316

[19] Zeng L Walsh M Guyatt GH Plasma exchange and glucocorticoid dosing for patients with ANCA-associated vasculitis: a clinical practice guideline. BMJ. 2022;376(1):e064597. (doi: 10.1136/bmj-2021-064597) 35217581

[20] Suzuki J Furuta S Kameoka Y Dynamics of scFv-targeted VAP2 correlating with IL-16, MIF and IL-1RA in ANCA-associated vasculitis. Microvasc Res. 2024;156(1):104720. (doi: 10.1016/j.mvr.2024.104720) 39127096

[21] Kong YZ Chen Q Lan HY. Macrophage migration inhibitory factor (MIF) as a stress molecule in renal inflammation. Int J Mol Sci. 2022;23(9):4908. (doi: 10.3390/ijms23094908) PMC9102975 35563296

[22] Lan HY. Role of macrophage migration inhibition factor in kidney disease. Nephron Exp Nephrol. 2008;109(3):e79 e83. (doi: 10.1159/000145463) 18663334

[23] Hao J Lv T Xu L Macrophage migration inhibitory factor is involved in antineutrophil cytoplasmic antibody-mediated activation of C5a-primed neutrophils. BMC Immunol. 2019;20(1):22. (doi: 10.1186/s12865-019-0306-z) PMC6598351 31248381

[24] Brown FG Nikolic-Paterson DJ Hill PA Urine macrophage migration inhibitory factor reflects the severity of renal injury in human glomerulonephritis. J Am Soc Nephrol. 2002;13(suppl 1):S7 S13. (doi: 10.1681/ASN.V13suppl_1s7) 11792756

[25] Kambas K Chrysanthopoulou A Vassilopoulos D Tissue factor expression in neutrophil extracellular traps and neutrophil derived microparticles in antineutrophil cytoplasmic antibody associated vasculitis may promote thromboinflammation and the thrombophilic state associated with the disease. Ann Rheum Dis. 2014;73(10):1854 1863. (doi: 10.1136/annrheumdis-2013-203430) 23873874

[26] Sun XJ Chen M Zhao MH. Thrombin contributes to anti-myeloperoxidase antibody positive IgG-mediated glomerular endothelial cells activation through SphK1-S1P-S1PR3 signaling. Front Immunol. 2019;10:237. (doi: 10.3389/fimmu.2019.00237) 30891029 PMC6413724

[27] Miao D Li DY Chen M Platelets are activated in ANCA-associated vasculitis via thrombin-PARs pathway and can activate the alternative complement pathway. Arthritis Res Ther. 2017;19(1):252. (doi: 10.1186/s13075-017-1458-y) PMC5688714 29141676

[28] Brandhofer M Hoffmann A Blanchet X Heterocomplexes between the atypical chemokine MIF and the CXC-motif chemokine CXCL4L1 regulate inflammation and thrombus formation. Cell Mol Life Sci. 2022;79(10):512. (doi: 10.1007/s00018-022-04539-0) PMC9468113 36094626

[29] Chen X Zhang S You R Renal damage and old age: risk factors for thrombosis in patients with ANCA-associated vasculitis. Thromb J. 2024;22(1):29. (doi: 10.1186/s12959-024-00593-9) PMC10953224 38509585

[30] Calandra T Bernhagen J Mitchell RA The macrophage is an important and previously unrecognized source of macrophage migration inhibitory factor. J Exp Med. 1994;179(6):1895 1902. (doi: 10.1084/jem.179.6.1895) 8195715 PMC2191507

[31] Bruchfeld A Carrero JJ Qureshi AR Elevated serum macrophage migration inhibitory factor (MIF) concentrations in chronic kidney disease (CKD) are associated with markers of oxidative stress and endothelial activation. Mol Med. 2009;15(3-4):70 75. (doi: 10.2119/molmed.2008.00109) 19081768 PMC2600496

[32] Musiał K Zwolińska D. Bone morphogenetic proteins (BMPs), extracellular matrix metalloproteinases inducer (EMMPRIN), and macrophage migration inhibitory factor (MIF): usefulness in the assessment of tubular dysfunction related to chronic kidney disease (CKD). J Clin Med. 2021;10(21):4893. (doi: 10.3390/jcm10214893) PMC8585016 34768412

[33] Linton MF Yancey PG Tao H HDL function and atherosclerosis: reactive dicarbonyls as promising targets of therapy. Circ Res. 2023;132(11):1521 1545. (doi: 10.1161/CIRCRESAHA.123.321563) 37228232 PMC10213997

[34] Madaudo C Bono G Ortello A Dysfunctional high-density lipoprotein cholesterol and coronary artery disease: a narrative review. J Pers Med. 2024;14(9):996. (doi: 10.3390/jpm14090996) PMC11432852 39338250

[35] M J L Badiger S Kadakol GS. Macrophage migration inhibitory factor gene polymorphism in acute coronary syndrome. Cureus. 2024;16(9):e69914. (doi: 10.7759/cureus.69914) PMC11495821 39439602

[36] Ahn JK Hwang J Choi CB Risk of acute myocardial infarction, stroke, and venous thromboembolism among patients with anti-neutrophil cytoplasmic antibody-associated vasculitis in South Korea: a nationwide population-based study. Jt Bone Spine. 2023;90(2):105498. (doi: 10.1016/j.jbspin.2022.105498) 36423779

[37] Goyal A Abbasi HQ Mashkoor Y Assessment of cardiovascular risk in patients with ANCA-associated vasculitis: a systematic review and meta-analysis. Int J Cardiol CardioVasc Risk Prev. 2024;23(1):200334. (doi: 10.1016/j.ijcrp.2024.200334) PMC11481614 39417001

[38] Zheng Z Wang Y Xie J The association between serum lipids at diagnosis and renal outcome in microscopic polyangiitis patients. PeerJ. 2025;13(1):e18839. (doi: 10.7717/peerj.18839) PMC11823655 39950045

[39] Kronbichler A Leierer J Shin JI Association of pulmonary hemorrhage, positive proteinase 3, and urinary red blood cell casts with venous thromboembolism in antineutrophil cytoplasmic antibody-associated vasculitis. Arthritis Rheumatol. 2019;71(11):1888 1893. (doi: 10.1002/art.41017) 31216123 PMC6899947

