# Erratum

**DOI:** 10.5152/ArchRheumatol.2026.00001

**Published:** 2026-07-01

In the original article authored by Lv T, Li Y, Xu L, Hao J, titled “Plasma  Macrophage Migration Inhibitory Factor as a Biomarker of Thromboinflammatory Dysregulation in Anti-Neutrophil Cytoplasmic Antibody-Associated Vasculitis” (Arch Rheumatol. 2026, 41, doi: 10.5152/ArchRheumatol.2026.25155) published in the second issue of Archives of Rheumatology, the institutional affiliations of the first and last authors were incorrectly presented due to an author oversight.

The corrected institution list is provided below:

^1^Division of Radiology, Department of Medicine, The Affiliated Hospital of Inner Mongolia Medical University, Hohhot, Inner Mongolia Autonomous Region, China

^2^Division of Nephrology, Department of Medicine, The Affiliated Hospital of Inner Mongolia Medical University, Hohhot, Inner Mongolia Autonomous Region, China

^3^Department of Medicine, Inner Mongolia Medical University, Hohhot, Inner Mongolia Autonomous Region, China

Upon receipt of the written request of the contributing authors, the Editorial Board reviewed the case and approved the affiliation list to be corrected. Subsequently, the affiliations were corrected, and the online version of the original article has been updated accordingly. You can access the revised version of this original article via the following link: https://archivesofrheumatology.org/index.php/pub/article/view/1553

